# Genetically programmed synthetic cells for thermo-responsive protein synthesis and cargo release

**DOI:** 10.1038/s41589-024-01673-7

**Published:** 2024-07-05

**Authors:** Carolina Monck, Yuval Elani, Francesca Ceroni

**Affiliations:** 1 Department of Chemical Engineering, Imperial College London, London, UK; 2 Imperial College Centre for Synthetic Biology, London, UK; 3 fabriCELL, Imperial College London, London, UK

**Keywords:** Synthetic biology, Synthetic biology

## Abstract

Synthetic cells containing genetic programs and protein expression machinery are increasingly recognized as powerful counterparts to engineered living cells in the context of biotechnology, therapeutics and cellular modelling. So far, genetic regulation of synthetic cell activity has been largely confined to chemical stimuli; to unlock their potential in applied settings, engineering stimuli-responsive synthetic cells under genetic regulation is imperative. Here we report the development of temperature-sensitive synthetic cells that control protein production by exploiting heat-responsive mRNA elements. This is achieved by combining RNA thermometer technology, cell-free protein expression and vesicle-based synthetic cell design to create cell-sized capsules able to initiate synthesis of both soluble proteins and membrane proteins at defined temperatures. We show that the latter allows for temperature-controlled cargo release phenomena with potential implications for biomedicine. Platforms like the one presented here can pave the way for customizable, genetically programmed synthetic cells under thermal control to be used in biotechnology.

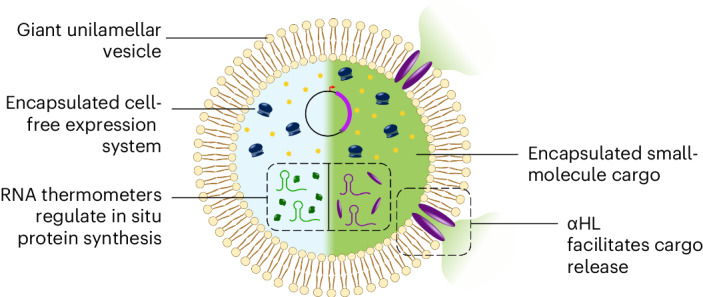

## Abstract (web summary)

RNA-based genetic regulation has been used to control protein translation in synthetic cells in response to the external temperature. RNA thermometers enable in situ protein expression above permissive temperatures. Controlled expression of the membrane pore α-hemolysin allows small-molecule cargo to be released in a temperature-dependent manner.

## Main

Synthetic cells are non-living biomimetic structures that have been engineered to possess features and capabilities like those of living biological cells, including protein synthesis, compartmentalization, motility, sense/response behaviors and communication^1^. As synthetic cells are built from the bottom up using molecular building blocks, researchers have enhanced control over the design of these systems and are unconstrained by the limitations associated with engineering living biological cells^2^. Away from the complex biochemical milieu of living cells, synthetic cells can be precisely programmed from first principles. They can also be functionalized with non-biological components, giving them enhanced functionality. These features make synthetic cells attractive as modular and flexible platforms, which can be purpose built for a diverse range of applications in medicine, biosensing, biomanufacturing and beyond^3–6^.

One of the benefits of synthetic biology is the ability to exploit the power of genetic regulation as a design principle. Genetic regulation has, until recently, been associated with top-down engineering of living cells, in applications from biosensing and biomanufacturing to therapeutics, such as chimeric antigen receptor T (CAR-T) cell therapy^7–10^. However, its use in synthetic cells is still in its infancy. To build a synthetic cell platform capable of genetic regulation, a cell-free protein expression (CFPE) system and a gene expression element must be encapsulated in a viable chassis (most commonly a lipid vesicle). CFPE systems contain the molecular machinery and biological building blocks for transcription and translation^11,12^, providing an easily customizable and efficient protein synthesis platform that has already proven useful for a range of commercial applications^13–16^. By using diverse synthetic gene circuits, control of gene expression can be achieved from simple activation switches^17^ to more complex feedback control loops^18^. Encapsulated CFPE systems are used to functionalize synthetic cells with gene regulation and expression capabilities, allowing the synthesis of proteins in a shielded compartment, distinct from the external environment.

When considering possible stimuli for genetically driven activation of synthetic cells, RNA-based regulators represent a useful toolbox as they do not siphon resources from the finite CFPE solution. Several such regulators have been demonstrated to be effective in synthetic cells, responding to a range of small-molecule stimuli^19–21^. However, although sensitivity to small molecules can be beneficial in bioprocessing settings, they are not always suitable as triggers for gene expression in therapeutic applications, where changes in local physiochemistry, especially at disease sites, can lead to off-target effects or failure of activation. Alternatively, physical stimuli, such as light, temperature, mechanical movement and magnetism, can be controlled and localized externally, minimizing this risk. There have already been examples of genetic constructs being used to build light-sensitive synthetic cells, responding to both ultraviolet and bioluminescence exposure^22,23^.

**Fig. 1:**
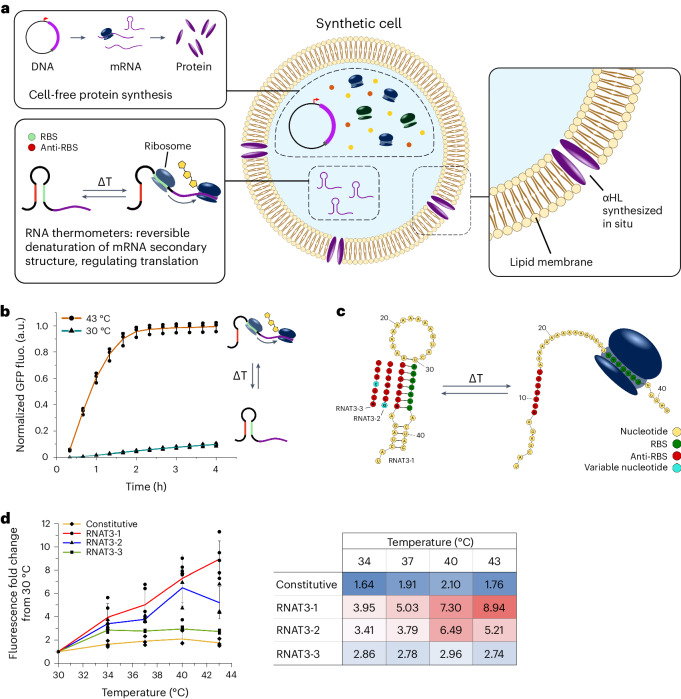
Designing a temperature-responsive synthetic cell. **a**, Synthetic cells are capable of in situ protein expression through encapsulation of CFPE systems (top left) that contain all the protein machinery and biological building blocks necessary for transcription and translation from a supplied DNA template. CFPE supports regulation of gene expression as leveraged in this work using RNATs (bottom left). RNATs control protein expression in response to temperature on the mRNA level by permitting or restricting access to the RBS. Bottom-up construction of synthetic cells also allows for the design of custom membrane compositions, including fluorescent lipids and integration of pores in the bilayer (right). In our synthetic cell system, the membrane pore αHL is synthesized in situ and self-inserts to allow cargo release. **b**, A suite of different thermometers (designed by Jia et al.^32^) was used to regulate dGFP expression. Each thermometer had single base-pair variations in the anti-RBS region, leading to a different minimal energy barrier and, thus, different activation temperature for expression. RNAT sequences are listed in Supplementary Table 1. **c**, Fluorescence recorded over 4 h from bulk CFPE reactions at 43 °C or 30 °C, expressing a cassette with dGFP under RNAT3-1 control. At 30 °C, the system is ‘off’ as the secondary structure of the RNAT is still intact, blocking ribosomal access to the RBS and inhibiting translation. Above the permissive temperature, at 43 °C, the system is ‘on’ as the secondary structure of the RNAT is denatured, freeing access to the RBS and allowing translation. *n* = 3 biological replicates for each temperature with the plotted line passing through mean values; error bars represent s.d. **d**, Thermometers were tested in a bulk CFPE system against a constitutively expressing dGFP construct at a range of different temperatures to assess their switching activity. Fold change of fluorescent signal at 43 °C versus 30 °C was plotted to compare activity across different RNATs (fold change = signal at 43 °C / signal at 30 °C). *n* = 3 biological replicates for each construct at each temperature, apart from RNAT3-1 where *n* = 5; plotted line passes through mean values, and error bars represent s.d. Supplementary Table 3 shows a heatmap of the mean fold changes of expression at each temperature, compared to 30 °C, along with standard deviations. fluo., fluorescence. Source data

One stimulus that is notably absent from the existing physical stimulus toolbox is temperature. Elevated temperature is associated with diseased states, classically with infection as well as additional pathologies, such as cancer^24^, making temperature a valuable control input. Temperature also has the potential to be an effective exogenous trigger, offering further spatial control to therapeutic application and delivery^25–27^. Examples of temperature-induced enzymatic conversions (and by extension magnetically induced ones)^28^ have been shown in cell-mimetic systems through the design of thermo-responsive compartments^29^, which can act as synthetic organelles interfaced with bacteria^6,30^. However, these systems were based solely on membrane biophysics phenomena and did not contain any gene expression regulation or protein synthesis functionality^31^. More recent work has since demonstrated temperature-dependent control of CFPE based on protein and RNA regulation^32^. Building on this, we demonstrate here the first example, to our knowledge, of an advanced temperature-responsive synthetic cell platform, capable of protein synthesis coupled with triggered cargo release (Fig. 1a). Specifically, we implemented RNA-based regulation of translation using RNA thermometers (RNATs) and demonstrate temperature-dependent in situ protein synthesis within synthetic cells. We then expanded this system to trigger synthesis of a membrane pore that can self-insert into the synthetic cell membrane, facilitating the release of pre-encapsulated small-molecule cargo. Our platform thus paves the way for similar systems to be used in controlled release applications, including therapeutics, using the physical stimulus of temperature.

## Results

### RNATs enable temperature-dependent protein expression

To achieve temperature-responsive control of gene expression, we adopted a suite of RNATs previously developed by Jia et al.^32^ (Supplementary Table 1). RNAT sequences contain a ribosome binding site (RBS) and complementary anti-RBS sequence (Fig. 1b); the sequence is located in the 5′ untranslated region between the promoter and the gene of interest. Upon transcription, the complementary bases in the RBS and anti-RBS sequences within the mRNA bind together, forming a hairpin. Different nucleotides in the anti-RBS will affect the binding affinity to the RBS, generating different minimal temperatures for activation. While the surrounding temperature remains below the thermometer activation threshold, there is insufficient free energy in the system to break the hairpin, and the ribosome cannot bind the RBS, inhibiting translation. Once the temperature rises beyond the threshold, the hairpin is denatured, allowing ribosome binding and subsequent translation. Native RNATs have been reported for bacteria and some eukaryotes in response to both increases and decreases in temperature, where they regulate cell adaptation of gene expression to changing environments^33–35^. Synthetic RNATs, such as those used in this work, have also been subsequently developed^36–39^.

DNA templates were constructed with a T7 promoter controlling the RNAT sequences upstream of a green fluorescent protein, dasherGFP (dGFP), which would be used as a proxy for thermometer activation (Supplementary Table 2). Validation of the RNAT switching behavior was first conducted in PURExpress, a commercial reconstituted CFPE kit based on *Escherichia* *coli* protein expression machinery with a T7 DNA polymerase^40^. We began by testing thermometer RNAT3-1 from Jia et al.^32^, following its activation over time at 43 °C compared to 30 °C (Fig. 1b). Two further RNAT sequences from the same paper were then added for testing (RNAT3-2 and RNAT3-3; Supplementary Table 1). These additional RNATs had single base variations in the anti-RBS region, leading to a different minimal energy barrier and, thus, different activation temperature for expression^32^. A constitutively expressed dGFP was also included as a control for the effect of temperature on CFPE activity. Assembled CFPE reactions were incubated at five different temperatures for 2 h, starting at 30 °C, when all RNATs would be ‘off’, inhibiting translation. Incrementally higher temperatures of 34 °C, 37 °C, 40 °C and 43 °C were used to assess the switching threshold of each thermometer. Endpoint readings of fluorescent intensity were taken and plotted as a fold change compared to expression at 30 °C (Fig. 1d and Supplementary Table 3). A positive correlation between dGFP expression and increasing temperature was observed across all RNATs, with a peak increase of 8.94-fold in expression for RNAT3-1 at 43 °C (Fig. 1d). In contrast to the relationship of increasing fold change with increasing temperature seen with the thermometers, the expression of constitutive dGFP (no RNA regulation) did not rise above 2.1-fold at any of the tested temperatures compared to 30 °C. This confirmed that the additional activity observed from the RNAT constructs was due to their switching capability rather than a thermodynamic effect of more efficient gene expression at higher temperatures.

### Development of an in situ expression platform in giant unilamellar vesicles

**Fig. 2:**
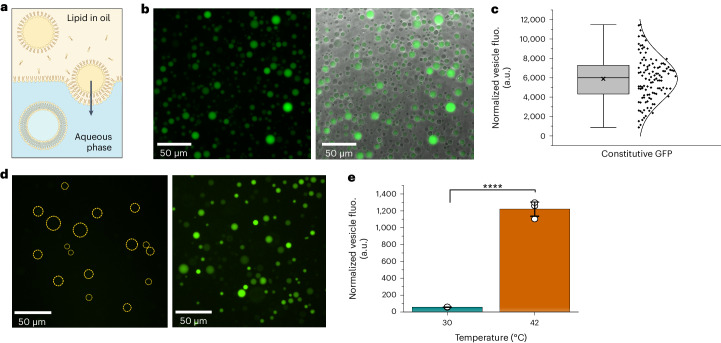
Formation of temperature-responsive synthetic cells. **a**, GUVs, the synthetic cell chassis, were formed using emulsion phase transfer: a lipid-in-oil emulsion containing aqueous droplets is driven through a lipid–aqueous interface to form a bilayer. The PURExpress CFPE system was included in the emulsion phase for encapsulation. **b**, Representative fluorescent and merged fluorescent–phase contrast images of synthetic cells after in situ expression of constitutive dGFP (not under RNAT control) at 42 °C. **c**, The distribution of fluorescence intensity of individual vesicles across the constitutively expressing population, normalized for background signal (Methods). *n* = 114 vesicles; box plot bounds the 25–75% data range with a central median line; **x** denotes mean signal; whiskers denote 1.5 times the interquartile range. Phase transfer does not allow precise control of encapsulation, leading to variation in expression efficiency (and, therefore, fluorescent signal intensity) across the population. **d**, Representative fluorescent images of synthetic cells containing dGFP under control of RNA3-1 after incubation for 2 h at 30 °C (left) and 42 °C (right). Dotted circles denote the location of fluorescing cells in the 30 °C sample. Cells incubated at 42 °C have stronger levels of dGFP expression compared to 30 °C due to the RNAT being switched ‘on’ at this higher temperature, enabling translation. **e**, Mean normalized signal intensity showed that cells incubated at 42 °C had significantly higher (21.03-fold) dGFP expression compared to those incubated at 30 °C (unpaired two-tailed *t-*test *P* = 3.91 × 10^−5^). *n* = 3 biological replicates with over 100 vesicles measured per replicate; **x** denotes mean signal; open circles denote mean signal of individual replicates. Full sample data can be found in Supplementary Fig. 4 and the Source Data file. fluo., fluorescence. Source data

Giant unilamellar vesicles (GUVs) were chosen to be the chassis for our synthetic cell system owing to their similarities in size with living cells (~1–50 µm diameter). GUVs can encapsulate a range of cargoes, from single-molecule dyes to complex protein mixtures^41^. Membrane composition can also be customized with different lipid ratios, including fluorescent labeling^41,42^ (Supplementary Fig. 1). We chose to use a composition of 100% 1-palmitoyl-2-oleoyl-glycero-3-phosphocholine (POPC), a commonly used phospholipid known to be biocompatible. The PURExpress CFPE system was used for in situ protein synthesis owing to its ease of use and compatibility with the GUV protocol for encapsulation. Synthetic cells were formed using emulsion phase transfer to encapsulate the PURExpress and relevant plasmid (Fig. 2a). Initially, synthetic cells containing a constitutive dGFP reporter were incubated at 42 °C to verify that temperature would not affect in situ protein expression (Fig. 2b). This was confirmed, although it was noted that expression was heterogeneous across the synthetic cell population (Fig. 2c). However, this was not unexpected and was previously described in similar systems^43–45^.

### Temperature-dependent protein expression in synthetic cells

After confirmation that we could successfully achieve gene expression in synthetic cells, we integrated RNAT control into the system for temperature-dependent expression. RNAT3-1 was selected as the candidate thermometer, having displayed the strongest fold change in bulk CFPE testing. Synthetic cells containing the PURExpress CFPE system and RNAT3-1 were formed, and, to maintain comparability, each sample was split for incubation at 30 °C or 42 °C for 2 h (Supplementary Fig. 2). Samples were then visualized using fluorescent microscopy (Fig. 2d, Supplementary Fig. 3 and Supplementary Video 1). Cells incubated at 42 °C showed a 21.03-fold increase in expression compared to 30 °C (Fig. 2e and Supplementary Fig. 4). Constitutively expressing synthetic cells showed only a 1.48-fold increase in expression across the same temperature shift (Supplementary Fig. 5), confirming that the increase seen in the RNAT3-1 cells was due to switching activity of the thermometer^46^.

### Temperature-triggered cargo release from synthetic cells

To advance the usability of our system in downstream applications where controlled cargo release is desirable, we investigated the integration of temperature response with expression of the pore protein α-hemolysin (αHL). αHL is a well-characterized pore-forming toxin secreted by *Staphylococcus aureus*^47^ with a pore diameter of 14–46 Å^48^, meaning that large proteins, such as GFP, are unable to pass through. Small molecules, however, including short peptides and nucleotide strands, can passively diffuse across^49,50^. αHL has been used extensively in synthetic biology and bottom-up synthetic cell work, enabling communication between compartments or providing an exogenous secretion system^51^. It is also an attractive choice of membrane pore for an autonomous synthetic cell system as the folded protein can self-insert into lipid bilayers without additional chaperones^52^. This is particularly valuable when synthesizing the pore in situ, as CFPE resources are finite. To this end, αHL was previously used in synthetic cells to facilitate cargo release in response to a small-molecule trigger^21^. To integrate this on-demand release capability into our synthetic cell platform, we engineered a suite of DNA templates containing the αHL gene under a T7 promoter and either constitutive (no RNAT) or temperature (RNAT3-1) control (Supplementary Table 2).

**Fig. 3:**
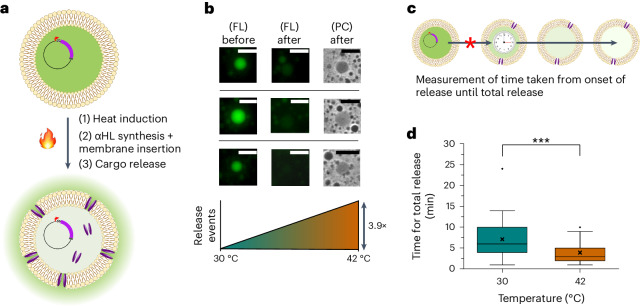
Integration of αHL for temperature-controlled cargo release. **a**, Schematic of the temperature-sensitive system, with αHL under control of the RNAT. Upon induction, the αHL is synthesized, and mature proteins form complexes that self-insert into the bilayer, allowing cargo release. **b**, Fluorescent and phase contrast images of individual synthetic cells taken at points throughout the timelapse from the sample incubated at 42 °C. The images show the level of fluorescent signal before (left column) and after (middle column) insertion of αHL, which causes the release of the fluorescent calcein cargo. The same vesicles are still visible in phase contrast images at the end of the timelapse (right column), confirming that the loss of fluorescent signal is due to a release event rather than destruction of the vesicles. The cell population incubated at 42 °C recorded 3.9-fold more release events than the 30 °C population. Scale bars, 20 µm. **c**,**d**, The time taken for vesicles to completely release their contents was measured to better understand the difference in kinetics at the two incubation temperatures: 30 °C (*n* = 34) or 42 °C (*n* = 112). **c**, Cargo release was measured from the first major drop in fluorescence until the cell signal matched the background fluorescence. **d**, The mean time taken for total release of cargo at 42 °C was significantly less than at 30 °C (unpaired two-tailed *t-*test *P* = 2.38 × 10^−7^). Box plot bounds the 25–75% data range with a central median line; **x** denotes mean signal; whiskers denote 1.5 times the interquartile range. FL, fluorescent; PC, phase contrast. Source data

Flow cytometry was used to initially characterize αHL-driven release. GUVs were formed with a cargo of calcein, a fluorescent small-molecule dye, and αHL protein was added externally. Upon self-insertion of αHL into the GUV membranes, the calcein was released via diffusion, and the vesicles no longer fluoresced (Fig. 3a and Supplementary Fig. 6). We then moved to engineering this capability into the synthetic cells using temperature-responsive in situ expression of αHL. Cells were formed with a cargo of PURExpress, the αHL gene under RNAT3-1 control and the calcein dye to track pore insertion through loss of fluorescence. After formation, cells were incubated at 30 °C or 42 °C for 30 min to allow gene expression before being moved to the microscope to monitor the dynamics of pore insertion. Samples were imaged every minute for 3 h to capture the release of fluorescent calcein cargo from individual cells, remaining at room temperature for the duration of the timelapse (Supplementary Video 2).

Crucially, cells that underwent release events were still visible in phase contrast imaging at the end of the timelapse (Fig. 3b). This confirmed that cells were releasing the calcein cargo through a pore mechanism rather than bursting, as can occur with osmotically imbalanced cargoes. A further control experiment was used to support this: cells with PURExpress and calcein cargo but without a plasmid for αHL expression were incubated at 42 °C and monitored under the same conditions for 3 h. The timelapses were used to quantify the level of cargo release at 30 °C compared to 42 °C as a proportion of the total population. Normalized for the basal level of release seen in the control, the 42 °C population had a 3.9-fold higher release activity compared to 30 °C (Fig. 3b). This lower fold change compared to dGFP expression is not unexpected given that we measured the binary event of calcein release from the vesicles rather than direct αHL amounts. Coupled with the known low-level expression from the thermometer at 30 °C (Supplementary Fig. 7), it is likely that at 30 °C, some cells may have sufficient expression to form αHL and allow calcein cargo release.

To further characterize the system, the kinetics of cargo release at different temperatures were also analyzed. Measurements were taken from the first major drop in recorded fluorescence, recording the time taken for vesicles to fully empty—that is, until the internal fluorescent signal matched that of the local background (Fig. 3c). Comparing the means of the two populations showed that cells at 42 °C released 1.8-fold faster than at 30 °C (Fig. 3d), with all cells incubated at 42 °C taking 10 min or less to undergo complete release. In contrast, nearly a quarter of the 30 °C population (23.5%) took over 10 min for complete release to occur. As the samples were at room temperature for the duration of the timelapse, we can be confident that this difference in release kinetics is due to the activated thermometers driving pore formation (more pores at higher temperature) rather than an effect of temperature on the speed of calcein diffusion. Together, these data show that at 42 °C, we see both more cells releasing, and with a faster rate of release across the population compared to 30 °C. Compounding the two measurements, the overall activity of the activated thermometer is 7.02-fold greater at 42 °C than at 30 °C.

## Discussion

Synthetic cells have great potential as bioinspired microdevices for applications in biomedicine, therapeutic delivery and beyond. Their modular and programmable nature facilitates logical engineering for manufacturing platforms, and their behaviors are being developed to increasingly match those of living cells. In this study, we adopted existing RNATs to develop synthetic cells capable of sensing the external environment, responding to the physical stimulus of temperature via translational control of gene expression. The triggered system can synthesize the membrane pore protein αHL in situ, allowing release of a small-molecule cargo.

The thermometers used in our synthetic cell system were originally designed in oil-based protocells^32^; by being able to exist in an aqueous environment, our membrane-bound synthetic cells bring the platform closer to biocompatibility. The in situ protein expression capabilities of our synthetic cells have also been validated for both soluble and membrane proteins. The latter, demonstrated using αHL, allows for controlled cargo release, with elevated temperature directly increasing the number of vesicles that release their content as well as the rate of release. Although synthetic cell systems using regulation at the mRNA level have been developed previously, to date these have all used chemical triggers^20,21^. The use of physical stimuli, such as temperature, for protein expression is a clear step forward as it can be applied with spatiotemporal control^53^, and temperature is a critical parameter in many biotechnological processes where synthetic cells are envisaged to have applications (for example, bioprocessing, bioproduction and biosensing). Elevated temperature has already been used as an exogenous trigger in a therapeutic setting to target areas for therapeutic action^32,54^. Temperature-responsive systems could, therefore, be used to control the timing and location for release of approved therapeutic molecules, increasing efficacy and widening the therapeutic window.

Our system can be considered an alternative to traditional methods of engineering thermally activated content release from membrane capsules, some of which are already being used in the clinic^55^. These normally depend on the breakdown of membranes using phase transitions and other biophysical phenomena^56,57^. Synthetic cell systems, on the other hand, allow the synthesis of the active ingredient on-site via CFPE. This subsequently provides more diverse options for regulation as genetic control can be multiplexed across many synthetic motifs, such as logic gates, oscillatory circuits, feedforward loops and signaling cascades—all of which have potential in biotechnological applications^58–60^. Leveraging both genetic control and customizable membrane components, synthetic cells can combine the best features of lipid nanoparticles and engineered cell technologies. Logic gates could be designed to link activation on a genetic level with responsive biophysical properties^61–63^. By tying each logic gate input to a precise biochemical context or exogenous signal, these multi-layer pathways can deliver tight and tuneable protein production at the correct time and in the correct location, reducing the likelihood of off-target effects. Although the RNATs described in these experiments show a significant fold change in GUVs when temperature is increased, a minimal level of basal expression was still observed at lower temperature (Supplementary Fig. 7). For our system to be adopted in downstream applications, such as the clinic, a tighter switch may be necessary. This is something that can be achieved through further optimization of the RNAT sequence design to reduce expression leakiness and increase the fold change of protein production upon activation.

Finally, when considering synthetic cells as autonomous therapeutic delivery vehicles, self-contained control systems that do not require synthesis of additional transcription factors—such as that displayed here—are of particular use. The translational control demonstrated in our work relies solely on the thermodynamic stability of the mRNA thermometer hairpins. The benefits of this are two-fold. First, the RNAT system does not require any additional regulatory proteins or co-factors to exert expression control^64^. This allows all CFPE resources to be directed to synthesis of the active compound rather than being siphoned off to produce a regulatory molecule. Second, while supplementing the CFPE mix with exogenous proteins before encapsulation could be a potential solution, each addition of exogenous molecules to a CFPE system necessitates a degree of re-optimization. This makes the process both time-consuming and resource-intensive. The RNAT system allows immediate and complete utilization of CFPE resources toward the active product while remaining flexible with respect to the downstream protein under regulation, as we have shown.

Synthetic cells are not living, do not evolve and do not need to perform auxiliary functions associated with keeping them alive. This represents a considerable advantage of synthetic cell-based technologies over engineered live cell technologies in clearing the regulatory hurdles toward clinical approval and industrial adoption. For synthetic cells to fulfil this potential, it is vital that the toolbox of available biosensors and engineering circuits continues to be expanded, increasing the sophistication of what we can achieve in synthetic cell specificity and programmability^60^. The individual components comprising synthetic cells will also have to be iterated upon to improve commercial viability. These include advances in the stability and shelf life of the GUV chassis, the uniformity of population size and expression efficiency and the lifespan of the encapsulated protein expression systems, to prolong activity and increase yields. More efficient manufacturing pipelines, such as those involving microfluidic technologies, will be of use in this^43,65^.

Our work demonstrates that it is possible to leverage the power of genetic regulation in a non-living chassis, paving the way for this platform to be used in downstream applications in therapeutics, biosensing and beyond. Coupled with responsive regulatory elements for an expanded repertoire of stimuli, this would drive synthetic cells toward biotechnological applications as viable competitors to engineered cells and traditional liposomal biotechnologies^4^.

## Methods

### Bacterial culture and molecular cloning

A plasmid containing dGFP under a T7 promoter was synthesized by DNA 2.0, containing an *E. coli* pJexpress 441 vector with T7 promoter and terminator sequences (cat. no. FP-03-441). Sequences for the RNA thermometers were obtained from previous published work by Jia et al.^32^. RNAT plasmids for use in PURExpress were constructed by PCR-mediated insertion of the short thermometer sequences via primers into the T7 dGFP plasmid (Supplementary Table 4). Ligated final products were used to transform chemically competent DH5α *E. coli*, which were plated on LB agar containing 100 μg ml^−1^ ampicillin and incubated at 37 °C overnight. Plasmids were recovered using a Miniprep Kit (QIAGEN), and correct clones were identified via sequencing. αHL plasmids were constructed via molecular cloning based on the constitutively expressing pT7-WT-H6 plasmid, kindly provided by the Bayley group (University of Oxford)^66^. A complete list of plasmids constructed in this study can be found in Supplementary Table 1.

### Cell-free protein synthesis

A PURExpress In Vitro Protein Synthesis Kit (New England Biolabs (NEB)) was used for cell-free protein synthesis. Samples were assembled following the NEB protocol using 250 ng of plasmid DNA and an additional 0.5 µl of murine RNAse inhibitor (NEB). For dynamic measurements of CFPE over time, samples were incubated at the necessary experimental temperature for 4 h in a CLARIOstar Plus plate reader (BMG Labtech), taking a reading of dGFP fluorescence every 20 min (λ_ex/em_ 470-15/515-20). For endpoint readings to calculate fold change in expression, samples were incubated at the necessary experimental temperature for 2 h in a thermocycler before taking an endpoint reading of dGFP fluorescence using the CLARIOstar Plus plate reader at λ_ex/em_ 470-15/515-20. Fold change was calculated using signal at 42 °C / signal at 30 °C.

### Vesicle formation

GUVs were formed using the phase transfer method. First, 2 mg of 100% POPC lipid (Avanti Polar Lipids) or 99 mol% POPC and 1 mol% biotin-labeled lipids (16:0 Biotinyl Cap PE, Avanti Polar Lipids) mixtures were prepared from chloroform stocks. A gentle stream of nitrogen gas was used to evaporate the chloroform, leaving a lipid film. Films were left overnight in a desiccator to ensure complete evaporation. Then, 1 ml of mineral oil was added to each sample, followed by 45–60 min in a bath sonicator at 40 °C to allow complete dissolution into a lipid solution. An Eppendorf containing 300 µl of 0.5 M glucose was made, onto which 120 µl of lipid solution was layered. This was left to sit while the inner phase was assembled. Next, 200 µl of the lipid solution was added to an empty Eppendorf. To this was added 20 µl of the aqueous cargo—calcein (0.5 mM), PURExpress or a mix of the two. Aqueous solutions contained either 0.5 M sugar (sucrose, encapsulating calcein only) or 0.3 M (sucrose or maltotriose, encapsulating PURExpress). PURExpress cargo was made up to 25 µl following the standard NEB protocol with 0.3 M sugar added through the spare volume capacity. For αHL experiments, 0.5 mM calcein was also added to the cargo solution. Then, 20 µl of the cargo solution was transferred to the lipid solution. The lipid-aqueous mixture was vortexed rapidly for 30 s to create a stable emulsion, which was gently pipetted into the second Eppendorf containing glucose and pre-layered lipid. Layered samples were then spun at 8.0 r.c.f. for 45 min. The supernatant was removed from spun Eppendorfs, ensuring that all the oil was removed and the GUV pellet was left undisturbed. The pellet was then resuspended in 200 µl of fresh glucose (0.5 M for calcein-containing and 0.8 M for PURExpress-containing vesicles). When encapsulating calcein only, if there was a large amount of free calcein remaining in the resuspended sample, a second spin would be carried out for 15 min at 8.0 r.c.f. before repeating the clean and resuspension.

### Vesicle incubation, visualization and analysis

After GUV formation, samples were split to incubate at different temperatures in parallel. Thermometer samples were incubated at 30 °C and 42 °C for 2 h. αHL experiments were incubated for 30 min at the relevant temperature before being transferred to the microscope where they remained at room temperature for the duration of the timelapse. dGFP timelapses were achieved by using biotin-labeled lipids and neutravidin-coated (1 mg ml^−1^) coverslips. GUVs were visualized using fluorescence microscopy on a Nikon eclipse Ti2-U inverted microscope with a CoolLED pE-300^white^ and a Nikon DS-Qi2 camera. Phase contrast was used to identify vesicles in bright-field, and a FITC-3540C filter cube was used for imaging dGFP/calcein fluorescence. For timelapse recordings of αHL samples, fluorescent images were taken every minute for 3 h. Repeat experiments were performed on a new Nikon eclipse Ti2-E inverted microscope with a D-LEDI and Prime BSI express camera using a 3-s exposure. For heated timelapse imaging, a Linkam PE100 Peltier heating stage was used with a T96 controller and water circulation pump. Images and timelapse files were analyzed using ImageJ and Excel to obtain fluorescence intensity, subtracting the local background signal for each GUV to generate a normalized dataset. Means of the normalized data were taken to give the representative population signal, and fold change was calculated using signal at 42 °C / signal at 30 °C. For analysis of all GUV signal intensity data, an unpaired two-tailed *t*-test assuming equal variance (α = 0.05) was used to determine the statistical significance of differences across datasets. *P* value: **** <0.0001, *** <0.0005, ** <0.005, * <0.05. The s.d. was calculated assuming the recorded values to be a sample of the whole population.

### Flow cytometry of vesicles

GUVs with encapsulated PURExpress were prepared following the methods above, with a lipid composition of POPC and 1 mol% 18:1 Liss Rhod PE to label the GUV membranes for robust particle detection on the flow cytometer. After formation, samples were split into three for incubation at the two experimental temperatures, and for running immediately on the flow to capture the calcein signal at *T* = 0. An Attune NxT Flow Cytometer (Thermo Fisher Scientific) was used for quantitative data collection during the calcein release assay (calcein was measured using a 488-nm blue laser and a 530/30 filter; rhodamine was measured using a 561-nm yellow laser and a 585/15 filter). Particle detection was gated according to physical and fluorescent parameters to ensure capture of the correct populations. Data analysis was performed using FlowJo software (BD Biosciences).

### Reporting summary

Further information on research design is available in the Nature Portfolio Reporting Summary linked to this article.

## Online content

Any methods, additional references, Nature Portfolio reporting summaries, source data, extended data, supplementary information, acknowledgements, peer review information; details of author contributions and competing interests; and statements of data and code availability are available at 10.1038/s41589-024-01673-7.

## Supplementary information

Supplementary Figs. 1–7, Tables 1–4 and descriptions for Supplementary Videos 1 and 2.

Reporting Summary

Supplementary Data 1 Source data for Supplementary Figs. 2 and 4–7.

Supplementary Data 2 Plasmid maps for all the constructs used in this study.

Supplementary Video 1a Microscopy fluorescent timelapses of synthetic cells expressing proteins in situ.

Supplementary Video 1b Microscopy fluorescent timelapses of synthetic cells expressing proteins in situ.

Supplementary Video 2 Microscopy fluorescent timelapses of synthetic cells expressing proteins in situ.

## Source data

Source Data Fig. 1 Plate reader measurements of dGFP expression in bulk CFPE at a range of different temperatures.

Source Data Fig. 2 Fluorescence measurements from microscopy images of GUVs expressing dGFP in situ (measured using Fiji).

Source Data Fig. 3 Fluorescence measurements from microscopy images of GUVs releasing fluorescent calcein cargo upon the insertion of αHL pores (measured using Fiji).

## Data Availability

All source data for datasets and figures presented in this manuscript are available as Supplementary Data files submitted with this manuscript. Constructs are available upon reasonable request from the corresponding author. Plasmid maps are available in the Supplementary Data files. Source data are provided with this paper.
