# Supplementary material for: Genetically programmed synthetic cells for thermo-responsive protein synthesis and cargo release
Source: Nat Chem Biol. 2024 Jul 5;20(10):1380–6. doi: 10.1038/s41589-024-01673-7 (PMC11427347; doi:10.1038/s41589-024-01673-7)
Supplement: Supplementary file 1 — Supplementary Figs. 1–7, Tables 1–4 and descriptions for Supplementary Videos 1 and 2. [file 41589_2024_1673_MOESM1_ESM.pdf]

nature chemical biology

Supplementary information

https://doi.org/10.1038/s41589-024-01673-7

Genetically programmed synthetic cells for thermo-responsive protein synthesis and cargo release

In the format provided by the authors and unedited

Supplementary Information

Supplementary Figures

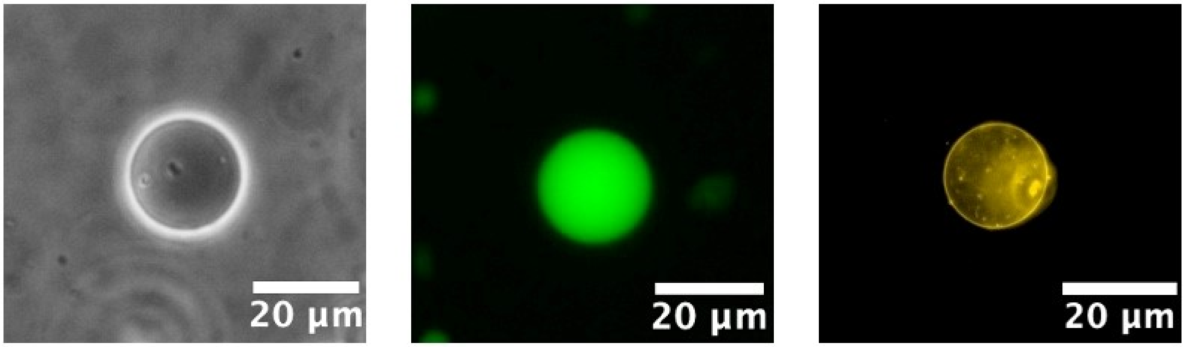

Supplementary Figure 1. **Giant unilamellar vesicles (GUVs).** From left to right, images show a representative GUV under phase contrast, with a fluorescent cargo (calcein), and with a fluorescently labelled membrane bilayer (rhodamine). N>10 GUV preparations with simple calcein cargo, giving similar phase contrast and calcein images. N>5 GUV preparations with rhodamine membrane labelling, producing similar images.

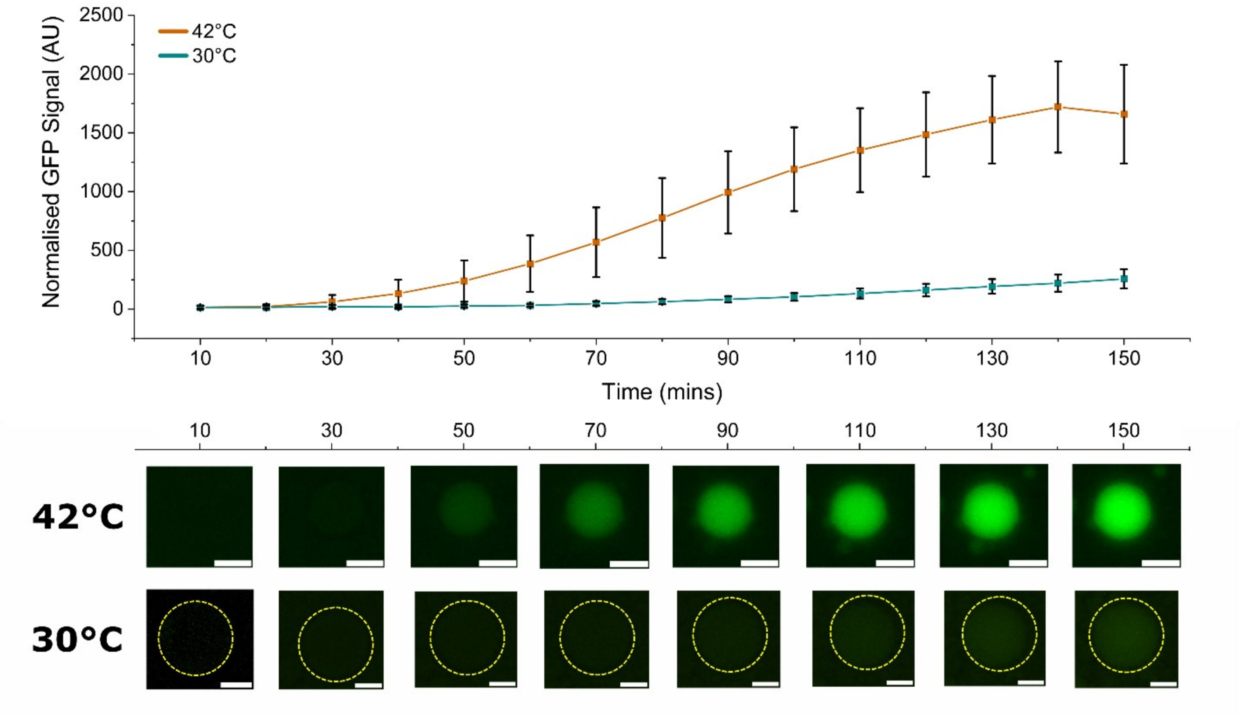

Supplementary Figure 2. dGFP expression over time from the RNAT3-1 sample at 30°C vs 42°C. Synthetic cells were formed carrying a plasmid for dGFP expression under the control of RNAT3-1. The sample was placed on a coverslip and mounted on a heating stage, where it was incubated for 2.5 hours at 30°C or 42°C. The heating stage was turned on at t=0, with images taken every 10 minutes for the duration of the timelapse. The normalised fluorescence of 32 vesicles was calculated per sample and plotted as mean signal +/- SD. At peak expression (t=140 mins), cells at 42°C were 7.78-fold more fluorescent than those at 30°C. Images of a single vesicle from each sample show the difference in fluorescence (scale bar = 10μm). The reduced fold-change compared to the endpoint values is attributed to the different methods of collection.

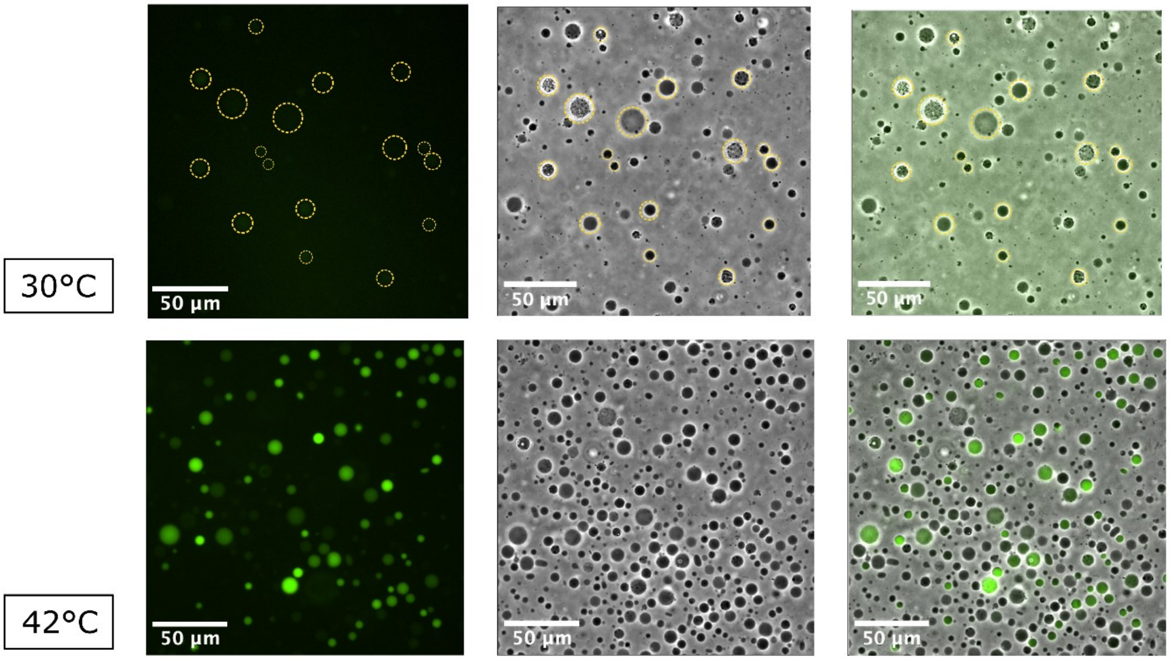

Supplementary Figure 3. Temperature-dependent expression of GFP in synthetic cells. Synthetic cells containing the gene for green fluorescent protein dGFP under control of the RNAT3-1 thermometer. From left to right, cells are shown in the fluorescent, phase contrast and merged channels. After preparation, synthetic cells were divided into two samples and incubated for two hours at 30°C (top) or 42°C (bottom). Yellow dashed circles indicate synthetic cells at 30°C which are fluorescent, but too weak to see.

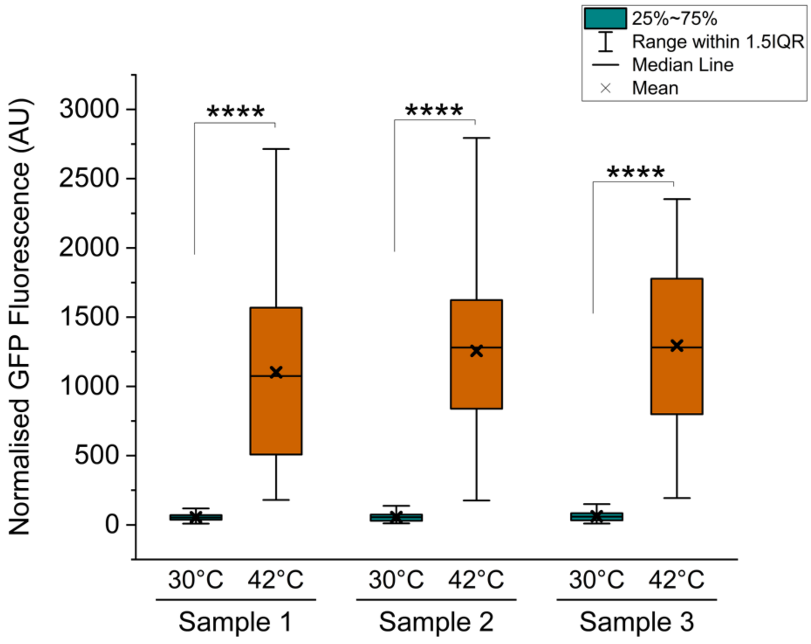

Supplementary Figure 4. Endpoint fluorescent data from temperature-dependent expression of GFP in synthetic cells. Synthetic cells were formed containing the gene for green fluorescent protein dGFP under control of the RNAT3-1 thermometer. After preparation, cells were divided into two samples and incubated for two hours at 30°C or 42°C. Cells were then visualised under the microscope and fluorescent readings were taken of >100 cells per sample. At 30 and 42°C respectively, Sample 1 n=171 and 138; Sample 2 n=100 and 129; Sample 3 n=108 and 116. All 3 samples had statistically significant increases in expression between 30°C and 42°C, utilising a two-tailed *t* test. p-values = 3.7219×10^{-64}, 3.0236×10^{-56} , 2.5463×10^{-57} for samples 1, 2 and 3 respectively. The mean fold-change of fluorescent signal from 30°C to 42°C was 21.03.

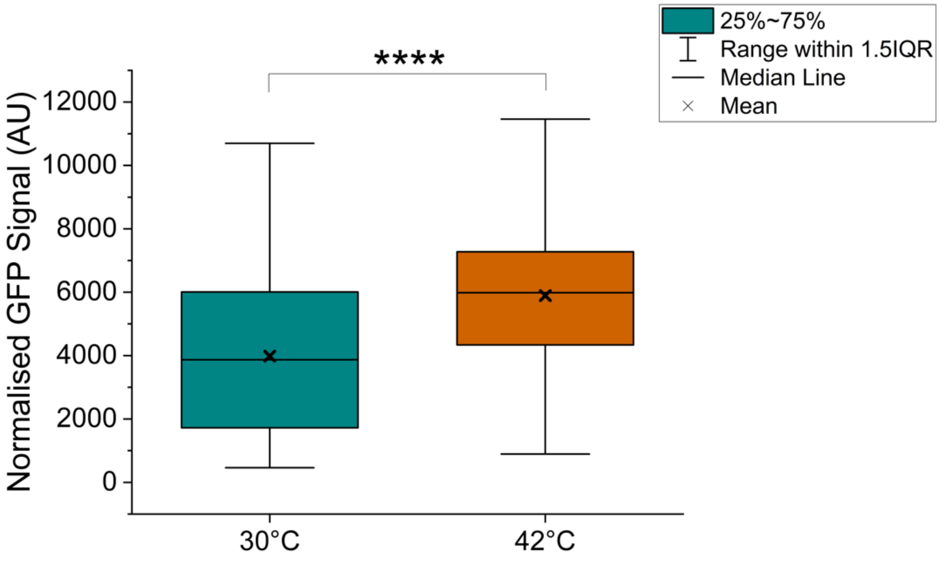

Supplementary Figure 5. Constitutive dGFP expression in synthetic cells at 30°C vs 42°C. Synthetic cells carrying a plasmid for constitutive expression of dGFP were formed. The sample was split and incubated for 2 hours at 30°C or 42°C before being imaged. Normalised fluorescence was calculated for n = 119 and n = 114 vesicles in the 30°C and 42°C populations respectively. The fold change in expression between the two temperatures was 1.48-fold, and a two tailed T-test determined the populations to be statistically distinct, with p-value = 7.7871×10^{-9}.

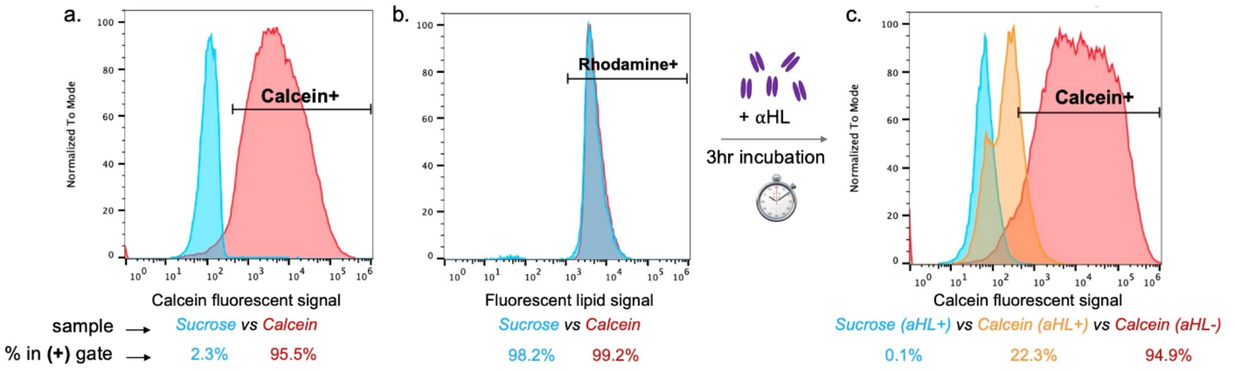

Supplementary Figure 6. α-hemolysin allows cargo release from GUVs. GUVs were formed containing either the fluorescent small molecule dye calcein, or sucrose (no CFPS). Sucrose-containing vesicles serve as a negative control for empty GUV fluorescence. The GUV membranes were also labelled with the fluorescent lipid Rhodamine (18:1 Liss Rhod PE) to facilitate particle detection in the flow cytometer. The two left hand graphs show **(a)** the fluorescent signal detected from the vesicle populations containing sucrose (blue) and calcein (red), before the addition of any external αHL protein. This was used to set the "Calcein+" gate which defined the boundaries of signal where vesicles containing calcein would fall. **(b)** the superimposed peaks of the sucrose- (blue) and calcein- (red) containing vesicles demonstrate that the fluorescent lipid profile of the two samples is virtually identical. Any difference in signal detected in the Calcein+ gate is therefore a measurement only of the encapsulated cargo, and not an artefact of membrane labelling. **(c)** following external addition of αHL to the samples, we can see that the calcein/αHL+ vesicles (orange) have lost 76.9% of the signal, compared to before incubation. The calcein/αHL- vesicles (red) prove that the shift is due to the addition of αHL, rather than the incubation time. The sucrose/ αHL+ vesicles (blue) confirm that the loss of signal is due to release via a pore, rather than disintegration of the vesicles, as the vesicles are still detectable across all samples.

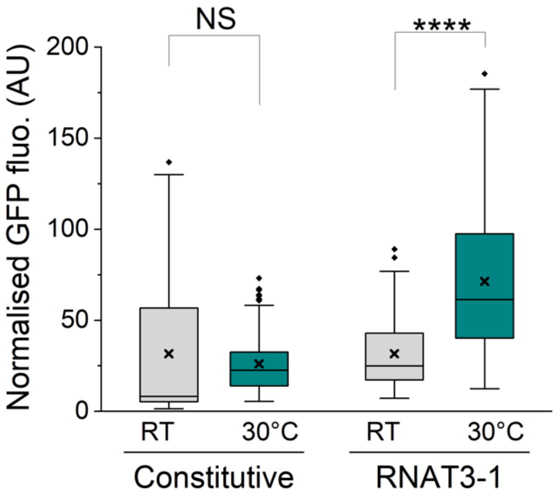

Supplementary Figure 7. Leakiness of the RNA thermometer. By comparing the change in expression between room temperature (RT) and 30°C for dGFP and under constitutive expression or RNAT3-1 control, it can be noted that the signal from the thermometer sample increases even with this low temperature. Since the difference in signal of constitutive GFP between the two temperatures is not statistically significant, we can assume that any change in signal is due to genuine expression activity rather than an artefact of more efficient CFPS expression at higher temperatures. The increase seen in the RNAT3-1 sample is therefore due to the thermometer having a degree of "leakiness", where a low level of translation still occurs despite being below the activation temperature of the thermometer. Samples were normalised by measuring the fluorescent intensity of images in ImageJ and subtracting the local background fluorescent signal. Statistical significance was calculated using an unpaired two tail *t*-test assuming equal variances, where p-values = 0.128 and 3.737×10^{-17} for the Constitutive and RNAT3-1 samples respectively. α=0.05 and p value < 0.001 statistical significance is denoted by ****. n = 113, 123 for the constitutive sample at RT and 30°C respectively and n = 109, 106 for the RNAT3-1 sample at RT and 30°C respectively. Box plot bounds the 25-75% data range with a central median line, **x** denotes mean signal; whiskers denote the 1.5 interquartile range.

Supplementary Tables

RNA thermometer	DNA Sequence
RNAT3-1	TACTAGAACTTTTTAAAAAAAAAAAGTACTAAGGAGTACTAG
RNAT3-2	TACTAGA**G**CTTTTTAAAAAAAAAAAGTACTAAGGAGTACTAG
RNAT3-3	TACTAGAACT**C**TTTAAAAAAAAAAAGTACTAAGGAGTACTAG

Supplementary Table 1. DNA sequences of the RNA thermometers. The anti-RBS region is underlined and variable bases between different thermometers are emboldened.

Plasmid	Sequence
T7 – **dasherGFP**	TAATACGACTCACTATAGGGGAATTGTGAGCGGATAACAATTCCCCTCTAGAAAT<br>AATTTTGTTTAACTTTTAGGAGGTAAAA**ATGACGGCATTGACGGAAGGTGCAAA**<br>**ACTGTTTGAGAAAGAGATCCCGTATATCACCGAACTGGAAGGCGACGTCGAAG**<br>**GTATGAAATTTATCATTAAAGGCGAGGGTACCGGTGACGCGACCACGGGTACC**<br>**ATTAAAGCGAAATACATCTGCACTACGGGCGACCTGCCGGTCCCGTGGGCAACC**<br>**CTGGTGAGCACCCTGAGCTACGGTGTTCAGTGTTTCGCCAAGTACCCGAGCCAC**<br>**ATCAAGGATTTCTTTAAGAGCGCCATGCCGGAAGGTTATACCCAAGAGCGTACC**<br>**ATCAGCTTCGAAGGCGACGGCGTGTACAAGACGCGTGCTATGGTTACCTACGA**<br>**ACGCGGTTCTATCTACAATCGTGTCACGCTGACTGGTGAGAACTTTAAGAAAGA**<br>**CGGTCACATTCTGCGTAAGAACGTTGCATTCCAATGCCCGCCAAGCATTCTGTAT**<br>**ATTCTGCCTGACACCGTTAACAATGGCATCCGCGTTGAGTTCAACCAGGCGTAC**<br>**GATATTGAAGGTGTGACCGAAAAACTGGTTACCAAATGCAGCCAAATGAATCG**<br>**TCCGTTGGCGGGCTCCGCGGCAGTGCATATCCCGCGTTATCATCACATTACCTAC**<br>**CACACCAAACTGAGCAAAGACCGCGACGAGCGCCGTGATCACATGTGTCTGGT**<br>**AGAGGTCGTGAAAGCGGTTGATCTGGACACGTATCAGTGA**
T7 – RNAT3-1 – **dasherGFP**	TAATACGACTCACTATAGGGGAATTGTGAGCGGATAACAATTCCCCTCTAGATAC<br>TAGAACTTTTTAAAAAAAAAAAGTACTAAGGAGTACTAG**ATGACGGCATTGACG**<br>**GAAGGTGCAAAACTGTTTGAGAAAGAGATCCCGTATATCACCGAACTGGAAGG**<br>**CGACGTCGAAGGTATGAAATTTATCATTAAAGGCGAGGGTACCGGTGACGCGA**<br>**CCACGGGTACCATTAAAGCGAAATACATCTGCACTACGGGCGACCTGCCGGTCC**<br>**CGTGGGCAACCCTGGTGAGCACCCTGAGCTACGGTGTTCAGTGTTTCGCCAAGT**<br>**ACCCGAGCCACATCAAGGATTTCTTTAAGAGCGCCATGCCGGAAGGTTATACCC**<br>**AAGAGCGTACCATCAGCTTCGAAGGCGACGGCGTGTACAAGACGCGTGCTATG**<br>**GTTACCTACGAACGCGGTTCTATCTACAATCGTGTCACGCTGACTGGTGAGAAC**<br>**TTTAAGAAAGACGGTCACATTCTGCGTAAGAACGTTGCATTCCAATGCCCGCCA**<br>**AGCATTCTGTATATTCTGCCTGACACCGTTAACAATGGCATCCGCGTTGAGTTCA**<br>**ACCAGGCGTACGATATTGAAGGTGTGACCGAAAAACTGGTTACCAAATGCAGC**<br>**CAAATGAATCGTCCGTTGGCGGGCTCCGCGGCAGTGCATATCCCGCGTTATCAT**<br>**CACATTACCTACCACACCAAACTGAGCAAAGACCGCGACGAGCGCCGTGATCAC**<br>**ATGTGTCTGGTAGAGGTCGTGAAAGCGGTTGATCTGGACACGTATCAGTGA**
T7 – RNAT3-2 – **dasherGFP**	TAATACGACTCACTATAGGGGAATTGTGAGCGGATAACAATTCCCCTCTAGATAC<br>TAGAGCTTTTTAAAAAAAAAAAGTACTAAGGAGTACTAG**ATGACGGCATTGACG**<br>**GAAGGTGCAAAACTGTTTGAGAAAGAGATCCCGTATATCACCGAACTGGAAGG**<br>**CGACGTCGAAGGTATGAAATTTATCATTAAAGGCGAGGGTACCGGTGACGCGA**

	**CCACGGGTACCATTAAAGCGAAATACATCTGCACTACGGGCGACCTGCCGGTCC<br>CGTGGGCAACCCTGGTGAGCACCCTGAGCTACGGTGTTCAGTGTTTCGCCAAGT<br>ACCCGAGCCACATCAAGGATTTCTTTAAGAGCGCCATGCCGGAAGGTTATACCC<br>AAGAGCGTACCATCAGCTTCGAAGGCGACGGCGTGTACAAGACGCGTGCTATG<br>GTTACCTACGAACGCGGTTCTATCTACAATCGTGTCACGCTGACTGGTGAGAAC<br>TTTAAGAAAGACGGTCACATTCTGCGTAAGAACGTTGCATTCCAATGCCCGCCA<br>AGCATTCTGTATATTCTGCCTGACACCGTTAACAATGGCATCCGCGTTGAGTTCA<br>ACCAGGCGTACGATATTGAAGGTGTGACCGAAAAACTGGTTACCAAATGCAGC<br>CAAATGAATCGTCCGTTGGCGGGCTCCGCGGCAGTGCATATCCCGCGTTATCAT<br>CACATTACCTACCACACCAAACTGAGCAAAGACCGCGACGAGCGCCGTGATCAC<br>ATGTGTCTGGTAGAGGTCGTGAAAGCGGTTGATCTGGACACGTATCAGTGA**
T7 – RNAT3-3 –<br>**dasherGFP**	TAATACGACTCACTATAGGGGAATTGTGAGCGGATAACAATTCCCCTCTAGATAC<br>TAGAACTCTTTAAAAAAAAAAAGTACTAAGGAGTACTAG**ATGACGGCATTGACG<br>GAAGGTGCAAAACTGTTTGAGAAAGAGATCCCGTATATCACCGAACTGGAAGG<br>CGACGTCGAAGGTATGAAATTTATCATTAAAGGCGAGGGTACCGGTGACGCGA<br>CCACGGGTACCATTAAAGCGAAATACATCTGCACTACGGGCGACCTGCCGGTCC<br>CGTGGGCAACCCTGGTGAGCACCCTGAGCTACGGTGTTCAGTGTTTCGCCAAGT<br>ACCCGAGCCACATCAAGGATTTCTTTAAGAGCGCCATGCCGGAAGGTTATACCC<br>AAGAGCGTACCATCAGCTTCGAAGGCGACGGCGTGTACAAGACGCGTGCTATG<br>GTTACCTACGAACGCGGTTCTATCTACAATCGTGTCACGCTGACTGGTGAGAAC<br>TTTAAGAAAGACGGTCACATTCTGCGTAAGAACGTTGCATTCCAATGCCCGCCA<br>AGCATTCTGTATATTCTGCCTGACACCGTTAACAATGGCATCCGCGTTGAGTTCA<br>ACCAGGCGTACGATATTGAAGGTGTGACCGAAAAACTGGTTACCAAATGCAGC<br>CAAATGAATCGTCCGTTGGCGGGCTCCGCGGCAGTGCATATCCCGCGTTATCAT<br>CACATTACCTACCACACCAAACTGAGCAAAGACCGCGACGAGCGCCGTGATCAC<br>ATGTGTCTGGTAGAGGTCGTGAAAGCGGTTGATCTGGACACGTATCAGTGA**
T7 – RNAT3-1 –<br>**αHL** – spacer – HisTag	TAATACGACTCACTATAGGGGAATTGTGAGCGGATAACAATTCCCCTCTAGATAC<br>TAGAACTTTTTAAAAAAAAAAAGTACTAAGGAGTACTAG**ATGGCAGATTCTGAT<br>ATTAATATTAAAACCGGTACTACAGATATTGGAAGCAATACTACAGTAAAAACA<br>GGTGATTTAGTCACTTATGATAAAGAAAATGGCATGCACAAAAAAGTATTTTAT<br>AGTTTTATCGATGATAAAAATCACAATAAAAAACTGCTAGTTATTAGAACAAAA<br>GGTACCATTGCTGGTCAATATAGAGTTTATAGCGAAGAAGGTGCTAACAAAAG<br>TGGTTTAGCCTGGCCTTCAGCCTTTAAGGTACAGTTGCAACTACCTGATAATGAA<br>GTAGCTCAAATATCTGATTACTATCCAAGAAATTCGATTGATACAAAAGAGTAT<br>ATGAGTACTTTAACTTATGGATTCAACGGTAATGTTACTGGTGATGATACAGGA<br>AAAATTGGCGGCCTTATTGGTGCAAATGTTTCGATTGGTCATACACTGAAATAT<br>GTTCAACCTGATTTCAAAACAATTTTAGAGAGCCCAACTGATAAAAAAGTAGGC<br>TGGAAAGTGATATTTAACAATATGGTGAATCAAAATTGGGGACCATACGATCG<br>AGATTCTTGGAACCCGGTATATGGCAATCAACTTTTCATGAAAACTAGAAATGG<br>TTCTATGAAAGCAGCAGATAACTTCCTTGATCCTAACAAAGCAAGTTCTCTATTA<br>TCTTCAGGGTTTTCACCAGACTTCGCTACAGTTATTACTATGGATAGAAAAGCAT<br>CCAAACAACAAACAAATATAGATGTAATATACGAACGAGTTCGTGATGATTACC<br>AATTGCATTGGACTTCAACAAATTGGAAAGGTACCAATACTAAAGATAAATGG<br>ACAGATCGTTCTTCAGAAAGATATAAAATCGATTGGGAAAAAGAAGAAATGAC<br>AAAT**CGTGGTTCGGGCTCATCTGGTGGCTCGAGTCACCATCATCATCACCACTAA<br>TGA

Supplementary Table 2. Sequences of all plasmids constructed in this work.

	30	34	37	40	43
**Constitutive**	1	1.64	1.91	2.10	1.76
StDev	0	0.27	0.38	0.63	0.32
**RNAT3-1**	1	3.95	5.03	7.30	8.94
StDev	0	1.18	1.48	2.06	1.57
**RNAT3-2**	1	3.41	3.79	6.49	5.21
StDev	0	0.38	0.28	1.57	1.39
**RNAT3-3**	1	2.86	2.78	2.96	2.74
StDev	0	0.19	0.06	0.14	0.13

Supplementary Table 3. Fold changes in GFP expression from 30°C from bulk CFPS testing of the RNA thermometers. Heat map indicates the range of fold changes across the different RNAT (standard deviations are not included in the heat map).

Name	Purpose	Sequence (5’-3’)
CM55	dasherGFP T7 universal RNAT downstream (FW)	AAAAAAAGTACTAAGGAGTACTAGATGACGGCATTGACGGAAG
CM56	dasherGFP T7 RNAT3 upstream (RV)	TTTTAAAGAGCTCTAGTATCTAGAGGGGAATTGTTATCC
CM57	dasherGFP T7 RNAT3-1 upstream (RV)	TTTTAAAAAGTTCTAGTATCTAGAGGGGAATTGTTATCC
CM58	dasherGFP T7 RNAT3-2 upstream (RV)	TTTTAAAAAGCTCTAGTATCTAGAGGGGAATTGTTATCC
CM59	dasherGFP T7 RNAT3-3 upstream (RV)	TTTTAAAGAGTTCTAGTATCTAGAGGGGAATTGTTATCC
CM61	dasherGFP backbone for αHL GFP operon (RV)	aaaaaTCTAGAGGGGAATTGTTATCCG
CM62	dasherGFP backbone for αHL GFP operon (FW) + XbaI	aaaaTCTAGAttaaAGGAGGTAAAAATGACGGCATTG
CM65	αHL universal RNAT integration FW	AAAAAAAGTACTAAGGAGTACTAGATGGCAGATTCTGATATTAATATTAAAACCGGT
CM66	αHL insert incl. HisTag + BamHI RV	aaaaGGATCCAAGCTTCATTAGTGGTG
CM67	pDasherGFP backbone for αHL insert (+BamHI) FW	aaaaGGATCCttaaCTAGCATAACCCCTTGGG

Supplementary Table 4. List of primers used in this work and their purposes. Lowercase bases represent spacer nucleotides.

Supplementary Videos

Supplementary Video 1. Timelapse of temperature-responsive synthetic cells expressing dGFP. Synthetic cells containing the gene for dGFP under control of the RNAT3-1 thermometer were imaged on a heating stage for 2 hours at either 30°C or 42°C. The heating stage was turned on at t=0, with images taken every 10 minutes for the duration of the timelapse.

Supplementary Video 2. Release of fluorescent cargo due to temperature-activated in situ synthesis of alpha hemolysin. Synthetic cells containing the fluorescent dye calcein, and the gene for alpha hemolysin under control of the RNAT3-1 thermometer. The sample has been incubated at 42°C for 30 minutes and then imaged for 3 hours, with pictures taken every minute to generate a timelapse. As the alpha hemolysin is synthesised and inserts into the membrane, release of the fluorescent calcein cargo is triggered leading to “disappearance” of the cells from the fluorescent image.
