# Supplementary material for: Genetically programmed synthetic cells for thermo-responsive protein synthesis and cargo release
Source: Nat Chem Biol. 2024 Jul 5;20(10):1380–6. doi: 10.1038/s41589-024-01673-7 (PMC11427347; doi:10.1038/s41589-024-01673-7)
Supplement: Supplementary file 2 — Reporting Summary [file 41589_2024_1673_MOESM2_ESM.pdf]

nature portfolio

Corresponding author(s): Francesca Ceroni

Last updated by author(s): 15 May 2024

Reporting Summary

Nature Portfolio wishes to improve the reproducibility of the work that we publish. This form provides structure for consistency and transparency in reporting. For further information on Nature Portfolio policies, see our Editorial Policies and the Editorial Policy Checklist.

Statistics

For all statistical analyses, confirm that the following items are present in the figure legend, table legend, main text, or Methods section.

n/a	Confirmed	
☐	☒	The exact sample size (*n*) for each experimental group/condition, given as a discrete number and unit of measurement
☐	☒	A statement on whether measurements were taken from distinct samples or whether the same sample was measured repeatedly
☐	☒	The statistical test(s) used AND whether they are one- or two-sided<br>*Only common tests should be described solely by name; describe more complex techniques in the Methods section.*
☐	☒	A description of all covariates tested
☐	☒	A description of any assumptions or corrections, such as tests of normality and adjustment for multiple comparisons
☐	☒	A full description of the statistical parameters including central tendency (e.g. means) or other basic estimates (e.g. regression coefficient) AND variation (e.g. standard deviation) or associated estimates of uncertainty (e.g. confidence intervals)
☐	☒	For null hypothesis testing, the test statistic (e.g. *F*, *t*, *r*) with confidence intervals, effect sizes, degrees of freedom and *P* value noted<br>*Give P values as exact values whenever suitable.*
☒	☐	For Bayesian analysis, information on the choice of priors and Markov chain Monte Carlo settings
☒	☐	For hierarchical and complex designs, identification of the appropriate level for tests and full reporting of outcomes
☒	☐	Estimates of effect sizes (e.g. Cohen's *d*, Pearson's *r*), indicating how they were calculated

Our web collection on statistics for biologists contains articles on many of the points above.

Software and code

Policy information about availability of computer code

Data collection	Bulk measurements were collected using fluorescence intensity reading in a plate reader. All vesicle data collection was carried out using microscopy. Flow cytometry data was collected using an Attune Flow Cytometer.
Data analysis	Plate reader fluorescence and vesicle signal data was analysed in Excel, using integrated functions for the calculation of means, standard deviations and t-tests. Vesicle data was analysed using Fiji software (Version 2.9.0/1.54b). Flow cytometry data was analysed using FlowJo software (Version 10.10)

For manuscripts utilizing custom algorithms or software that are central to the research but not yet described in published literature, software must be made available to editors and reviewers. We strongly encourage code deposition in a community repository (e.g. GitHub). See the Nature Portfolio guidelines for submitting code & software for further information.

Data

Policy information about availability of data

All manuscripts must include a data availability statement. This statement should provide the following information, where applicable:

- Accession codes, unique identifiers, or web links for publicly available datasets
- A description of any restrictions on data availability
- For clinical datasets or third party data, please ensure that the statement adheres to our policy

All source data for data sets and figures presented in this manuscript are available on Figshare and the provided Source Data file. Constructs are available upon request to the corresponding author.

Human research participants

Policy information about studies involving human research participants and Sex and Gender in Research.

Reporting on sex and gender	No human research participants were used in this research.
Population characteristics	n/a
Recruitment	n/a
Ethics oversight	n/a

Note that full information on the approval of the study protocol must also be provided in the manuscript.

Field-specific reporting

Please select the one below that is the best fit for your research. If you are not sure, read the appropriate sections before making your selection.

☒ Life sciences ☐ Behavioural & social sciences ☐ Ecological, evolutionary & environmental sciences

For a reference copy of the document with all sections, see nature.com/documents/nr-reporting-summary-flat.pdf

Life sciences study design

All studies must disclose on these points even when the disclosure is negative.

Sample size	Sample sizes were chosen to provide a representative sample of the overall population. This was informed by sample sizes used in existing literature which are accepted as providing representative coverage of population behaviours.
Data exclusions	For all GUV data, only GUVs greater than 5 microns in diameter were counted.
Replication	All bulk experiments were repeated with n>=3 biological replicates. All GUV data was accrued by measuring the maximum feasible number of GUVs for a given sample, to account for technical and biological variation. Not all experiments were successful, determined by the level of expression achieved from known control constructs. Only successful experiments were included in the n>=3 replicates.
Randomization	Randomisation was not necessary because selection bias was not present in the reported experiments; all samples were prepared following the same methods, using the same materials, and compared samples were derived from the same batch where relevant and possible.
Blinding	Blinding was not necessary as experimental outcomes were not subject to bias based on knowledge of conditions from experimenters.

Reporting for specific materials, systems and methods

We require information from authors about some types of materials, experimental systems and methods used in many studies. Here, indicate whether each material, system or method listed is relevant to your study. If you are not sure if a list item applies to your research, read the appropriate section before selecting a response.

Materials & experimental systems

n/a	Involved in the study	
☒	☐	Antibodies
☒	☐	Eukaryotic cell lines
☒	☐	Palaeontology and archaeology
☒	☐	Animals and other organisms
☒	☐	Clinical data
☒	☐	Dual use research of concern

Methods

n/a	Involved in the study	
☒	☐	ChIP-seq
☐	☒	Flow cytometry
☒	☐	MRI-based neuroimaging

Flow Cytometry

Plots

Confirm that:

☒ The axis labels state the marker and fluorochrome used (e.g. CD4-FITC).

☒ The axis scales are clearly visible. Include numbers along axes only for bottom left plot of group (a 'group' is an analysis of identical markers).

☒ All plots are contour plots with outliers or pseudocolor plots.

☒ A numerical value for number of cells or percentage (with statistics) is provided.

Methodology

Sample preparation	GUVs were formed containing either the fluorescent small molecule dye calcein, or sucrose (no Cell Free Protein Synthesis extract was used for any flow cytometry data). Sucrose-containing vesicles serve as a negative control for empty GUV fluorescence. The GUV membranes were also labelled with the fluorescent lipid Rhodamine (18:1 Liss Rhod PE) to facilitate particle detection in the flow cytometer. Following formation, samples were split into 3 for incubation at the two experimental temperatures, and for running immediately on the flow to capture the calcein signal at T=0.
Instrument	Attune Nxt Flow Cytometer (ThermoFisher)
Software	FlowJo was used for all data visualisation and analysis
Cell population abundance	approximately >95% of the sample was retained following Rhodamine gating.
Gating strategy	Rhodamine (membrane label) was used to gate for correct identification of GUVs. Sucrose-containing vesicles were used as a negative control to determine the lower bound of the calcein gate.

☒ Tick this box to confirm that a figure exemplifying the gating strategy is provided in the Supplementary Information.
